# Supplementary material for: Framework for feature selection of predicting the diagnosis and prognosis of necrotizing enterocolitis
Source: PLoS One. 2022 Aug 19;17(8):e0273383. doi: 10.1371/journal.pone.0273383 (PMC9390903; doi:10.1371/journal.pone.0273383)
Supplement: S1 Table — (DOCX) [file pone.0273383.s001.docx]

| All variables/features | Description of different features |
| --- | --- |
| **perinatal characteristics** |  |
| Sex | dichotomous variable: male and female. |
| GA | continuous variable, gestational age at birth. |
| BW | continuous variable, birth weight. |
| BW for GA | categorical variable, it is divided into three categories: small-for-gestational-age, appropriate-for-gestational-age and large-for-gestational-age. |
| Gravida | categorical variable. |
| Para | categorical variable. |
| Multiple births | categorical variable, it is divided into three categories: singles, twins and triplets. |
| Apgar 1-min | continuous variable, range 0-10. |
| Apgar 5-min | continuous variable, range 0-10. |
| Birth asphyxia | dichotomous variable: Yes or No. |
| Fetal distress | dichotomous variable: Yes or No. Fetal distress meets the diagnostic criteria of obstetrics and gynecology. |
| Neonatal resuscitation | dichotomous variable: whether to resuscitate according to the neonatal resuscitation algorithm. |
| Delivery mode | dichotomous variable: vaginal delivery and caesarean section. |
| Mother’s age | continuous variable, the mother’s age at birth |
| PPROM  PPROM-birth interval | dichotomous variables, preterm premature rupture of membranes.  continuous variable |
| Oligohydramnios | dichotomous variable: Yes or No. It was identified by the following methods: the four- quadrant amniotic fluid index (AFI), single deepest pocket (SDP), and two diameter pocket. it can be defined as <5%, <5cm (AFI), and <2cm (SDP). |
| Meconium amniotic fluid | dichotomous variable: Yes or No. |
| Placenta abnormalities | dichotomous variable: normal placentation and abnormal placentation. Placental abnormalities include placenta accrete, placenta previa, abruptio placenta, etc. |
| HELLP syndrome | dichotomous variable, the mother was diagnosed with HELLP syndrome during pregnancy |
| Hypertension | dichotomous variable, the mother was diagnosed with hypertensive disorders in pregnancy or had hypertension before pregnancy |
| GDM | dichotomous variable, gestational diabetes mellitus. The mother was diagnosed with GDM during pregnancy, GDM meets the diagnostic criteria established by the American Diabetes Association in 2013. |
| **Clinical features prior clinical onset** |  |
| ***Clinical diagnosis*** |  |
| PDA | dichotomous variable, patent ductus arteriosus. |
| Closing the catheter medication | dichotomous variable: Yes or No. |
| Drug name | categorical variable, using of drugs to close the arterial catheter. It is divided into three categories: no drug use, using acetaminophen, using Ibuprofen. |
| IVH | dichotomous variable, intraventricular hemorrhage. |
| Infectious diseases  CMV infection  Congenital syphilis  Urinary tract infection  Pneumonia  Early onset sepsis  Late onset sepsis  Other infection | dichotomous variables: Yes or No. If yes, select at least one of the following infectious diseases. |
| ***Medication*** |  |
| Early use of antibiotics | dichotomous variable, commencement of antibiotics within 3 postnatal days. |
| Duration of early antibiotic treatment | continuous variable. |
| PS | dichotomous variable, pulmonary surfactant use. |
| caffeine | dichotomous variable, caffeine use. |
| Corticosteroids | dichotomous variable, corticosteroids use. |
| Inotropic | dichotomous variable, inotropic use. |
| Anemia-RBC transfusion | categorical variable, it is divided into three categories: not anemia, anemia-not transfusion and anemia-transfusion. Neonatal anemia was defined as a central venous hematocrit < 39% based on the College of American Pathologists Neonatal Red Blood Cell Transfusion Guidelines. |
| RBC transfusion 72 hours prior clinical onset | dichotomous variable: Yes or No. |
| Total number of RBC transfusions | continuous variable. |
| ***Clinical operations*** |  |
| MV | dichotomous variable: Yes or No. Mechanical ventilation. |
| Umbilical Arteriovenous Catheter | dichotomous variable: Yes or No. |
| PICC | dichotomous variable, peripherally inserted central venous catheter. |
| ***Feeding strategies*** |  |
| Enteral nutrition start | categorical variable, it is divided into three categories: Slow, Medium, Quick. Slow, never started or started later than postnatal day 4; Medium, started on postnatal day 3 or 4; Quick, started within postnatal day 2. |
| Days on enteral nutrition | continuous variable. |
| Feeding volume at NEC onset | continuous variable. |
| Daily milk increment | dichotomous variable: slow and quick. Slow, the daily milk increment is less than 20 ml per kilogram of body weight until reaching full feeding volumes; quick, more than 20 ml per kilogram of body weight. |
| Type of milk | categorical variable, it is divided into three categories: human milk, formula milk and combination. |
| HMF | dichotomous variable, human milk fortifier. |
| Probiotics | dichotomous variable: Yes or No. |
| **Clinical features at clinical onset** |  |
| \| Postnatal age at clinical onset \|  \| \| --- \| --- \| | continuous variable |
| Corrected GA at clinical onset | continuous variable |
| ***Clinical manifestations*** | dichotomous variables: Yes or No. |
| Gastric residual | pre-feeding residuals ≥ 30% of the feeding volume |
| Emesis |  |
| Bloody stools |  |
| Abdominal distension |  |
| Signs of peritoneal irritation |  |
| Bowel sound attenuation |  |
| Fever |  |
| Temperature instability |  |
| Apnea |  |
| Drowsiness |  |
| Tachycardia | Heart rate＞160/min |
| Acidosis | pH value＜7.3 and pH site is arterial or when bicarbonate value＜16 |
| DIC | dichotomous variables, disseminated intravascular coagulation. |
| **Laboratory parameters** | blood routine results |
| WBC at birth |  |
| NEUT% at birth |  |
| LY% at birth |  |
| MO% at birth |  |
| NEUT# at birth |  |
| LY# at birth |  |
| MO# at birth |  |
| RBC at birth |  |
| HGB at birth |  |
| HCT at birth |  |
| MCV at birth |  |
| MCH at birth |  |
| RDW at birth |  |
| PLT at birth |  |
| PCT at birth |  |
| MPV at birth |  |
| PDW at birth |  |
| WBC at clinical onset (t0) |  |
| NEUT% at clinical onset (t0) |  |
| LY% at clinical onset (t0) |  |
| MO% at clinical onset (t0) |  |
| NEUT# at clinical onset (t0) |  |
| LY# at clinical onset (t0) |  |
| MO# at clinical onset (t0) |  |
| RBC at clinical onset (t0) |  |
| HGB at clinical onset (t0) |  |
| HCT at clinical onset (t0) |  |
| MCV at clinical onset (t0) |  |
| MCH at clinical onset (t0) |  |
| RDW at clinical onset (t0) |  |
| PLT at clinical onset (t0) |  |
| PCT at clinical onset (t0) |  |
| MPV at clinical onset (t0) |  |
| PDW at clinical onset (t0) |  |
| ***Laboratory values change*** | Percentage change of each indicator at clinical onset compared with those at birth |
| WBC change | （WBC at clinical onset -WBC at birth）/ WBC at birth |
| NEUT% change | （NEUT% at clinical onset - NEUT% at birth）/ NEUT% at birth |
| LY% change | （LY% at clinical onset - LY% at birth）/ LY% at birth |
| MO% change | （MO% at clinical onset - MO% at birth）/ MO% at birth |
| NEUT# change | （NEUT# at clinical onset - NEUT# at birth）/ NEUT# at birth |
| LY# change | （LY# at clinical onset - LY# at birth）/ LY# at birth |
| MO# change | （MO# at clinical onset - MO# at birth）/ MO# at birth |
| RBC change | （RBC at clinical onset -RBC at birth）/ RBC at birth |
| HGB change | （HGB at clinical onset - HGB at birth）/ HGB at birth |
| HCT change | （HCT at clinical onset - HCT at birth）/ HCT at birth |
| MCV change | （MCV at clinical onset - MCV at birth）/ MCV at birth |
| MCH change | （MCH at clinical onset - MCH at birth）/ MCH at birth |
| RDW change | （RDW at clinical onset - RDW at birth）/ RDW at birth |
| PLT change | （PLT at clinical onset - PLT at birth）/ PLT at birth |
| PCT change | （PCT at clinical onset - PCT at birth）/ PCT at birth |
| MPV change | （MPV at clinical onset - MPV at birth）/ MPV at birth |
| PDW change | （PDW at clinical onset - PDW at birth）/ PDW at birth |
