# Supplementary material for: Framework for feature selection of predicting the diagnosis and prognosis of necrotizing enterocolitis
Source: PLoS One. 2022 Aug 19;17(8):e0273383. doi: 10.1371/journal.pone.0273383 (PMC9390903; doi:10.1371/journal.pone.0273383)
Supplement: S2 Table — (DOCX) [file pone.0273383.s002.docx]

|  | **Parameter** | **value** |
| --- | --- | --- |
| **mRMR** | selection method | MIQ |
|  | k | 30 |
| **ReliefF** | metric | manhattan |
|  | k | 3 |
| **GA** | estimator | SVC |
|  | cv | 10 |
|  | scoring | roc_auc |
|  | max_features | 30 |
|  | n_population | 1200 |
| **BSO** | flip | 5 |
|  | nBees | 10 |
|  | maxIteration | 10 |
|  | localIteration | 10 |
| **RFE** | estimator | SVC |
|  | n_features_to_select | 32 |
| **LASSO** | alphas | 0.017 |
| **Ridge** | alphas | 503.15 |
